# Topology of Innovation Spaces in the Knowledge Networks Emerging through Questions-And-Answers

**DOI:** 10.1371/journal.pone.0154655

**Published:** 2016-05-12

**Authors:** Miroslav Andjelković, Bosiljka Tadić, Marija Mitrović Dankulov, Milan Rajković, Roderick Melnik

**Affiliations:** 1 Institute of Nuclear Sciences, Vinča, University of Belgrade, Belgrade, Serbia; 2 Department of Theoretical Physics, Jožef Stefan Institute, Ljubljana, Slovenia; 3 Scientific Computing Laboratory, Institute of Physics Belgrade, University of Belgrade, Zemun-Belgrade, Serbia; 4 MS2Discovery Interdisciplinary Research Institute, M^2^NeT Laboratory and Department of Mathematics, Wilfrid Laurier University, Waterloo, ON, Canada; 5 BCAM–Basque Center for Applied Mathematics, E48009 Bilbao, Basque Country–Spain; University of Maribor, SLOVENIA

## Abstract

The communication processes of knowledge creation represent a particular class of human dynamics where the expertise of individuals plays a substantial role, thus offering a unique possibility to study the structure of knowledge networks from online data. Here, we use the empirical evidence from questions-and-answers in mathematics to analyse the emergence of the network of knowledge contents (or tags) as the individual experts use them in the process. After removing extra edges from the network-associated graph, we apply the methods of algebraic topology of graphs to examine the structure of higher-order combinatorial spaces in networks for four consecutive time intervals. We find that the ranking distributions of the suitably scaled topological dimensions of nodes fall into a unique curve for all time intervals and filtering levels, suggesting a robust architecture of knowledge networks. Moreover, these networks preserve the logical structure of knowledge within emergent communities of nodes, labeled according to a standard mathematical classification scheme. Further, we investigate the appearance of new contents over time and their innovative combinations, which expand the knowledge network. In each network, we identify an innovation channel as a subgraph of triangles and larger simplices to which new tags attach. Our results show that the increasing topological complexity of the innovation channels contributes to network’s architecture over different time periods, and is consistent with temporal correlations of the occurrence of new tags. The methodology applies to a wide class of data with the suitable temporal resolution and clearly identified knowledge-content units.

## Introduction

The knowledge creation through online social interactions represents an emerging area of increased interest both for technological advances and the society [1] where the collective knowledge is recognised as a social value [2–4]. Recently studied examples include the knowledge accumulation in systems with direct questions-and-answers [5], crowdsourcing scientific knowledge production [6, 7] and scientific discovery games [8]. Similar phenomena can be observed in business/economics-associated online social networking [9–11]. On the other hand, the study of the collective knowledge creation opens new topics of research interests. In particular, it provides ground to examine a novel type of collective dynamics in social systems in which each actor possesses certain limited expertise. In the course of the collaborative social efforts to solve a problem, such as communications through questions-and-answers that we consider here, the tacit knowledge and the expertise of individual actors are externalised and dynamically shared with other participants who take part in the process. When a systematic tagging applies to the shared cognitive contents, the process leads to an explicit knowledge [3] as the output value (the network of knowledge contents), from which others can learn. Furthermore, the dynamics underlying knowledge creation exemplifies multi-scale phenomena related to the cognitive recognition, which may occur in a wider class of systems, social, biological and physical [17].

By the nature of the underlying stochastic processes, the knowledge networks that emerge through the collaborative social endeavours necessarily reflect the expertise and the activity patterns of the involved participants. Furthermore, these networks tend to capture the logical relationship among the used cognitive contents as it resides in the mind of each participating individual. In this regard, these networks substantially differ from the commonly studied knowledge networks, which are produced in ontological initiatives [12–14] such as those from the online bibliographic data and Wikipedia, or the mapping citation relationships between journal articles [15], to name a few. Also, the stochastic process of knowledge creation through questions and answers are different from the spreading dynamics of scientific memes, whose inheritance patterns are identified in citation networks [16].

In recent work [5], we have shown that the knowledge creation by questions-and-answers involve two-scale dynamics, in which the constitutive social and cognitive elements (individual experts or actors and the knowledge contents that they use) interact and influence each other on the original scale. This complex system evolves in a self-organised manner leading to the emergence of socio-technological structures where the involved actors share the accumulated knowledge. These structures are visualised as communities on the related bipartite network of actors and their artefacts [5]. Furthermore, the advance of innovation in this process, which builds on the expertise of the involved participants, leads to the expansion of the knowledge space by adding new cognitive contents. The central question for the research and applications of the collective knowledge creation is how these stochastic processes work and potentially can be controlled to converge towards the desired outcome. Furthermore, what is the structure of the emergent knowledge that can be used by others?

A part of the answer relies on the structure of the networks, co-evolving with the knowledge-sharing processes among the actors possessing the required expertise. In [5] the empirical data from the Stack Exchange site Mathematics (http://math.stackexchange.com/) were downloaded and analysed, as a prototypal example. The sequence of events in the process of questions-and-answers (Q&A) suitably maps onto a growing bipartite network of actors, as one partition, and their questions and answers, as another partition. The emergent communities on these networks have been identified, consisting of the involved actors and the connected questions-and-answers. As a rule, in each community a dominant actor is found, representing an active user with a broad expertise. The knowledge elements of each question are specified according to the standard mathematical classification scheme by one to five tags (for instance, “functional analysis”, “general topology”, “differential geometry”, “abstract algebra”, “algebraic number theory”). Consequently, the expertise of the actor can be specified as a combination of tags that the actor had frequently used. Assuming that a minimal matching applies among the actor’s expertise and the contents of the answered question, and using theoretical modelling based on the empirical data, it was shown [5] that the emergent communities and the knowledge that they share strongly depend on the population of the involved experts and their activity patterns.

In this work, using the same empirical dataset, our focus is on the networks of cognitive elements (tags) that emerge in these processes with questions-and-answers. Different from the aforementioned bipartite networks, these emergent knowledge networks contain subelements of both partitions, namely, knowledge contents of questions as well as a measure of the users’ expertise. Such networks, supported by the current information and computer technology (ICT) systems, embody the collective knowledge that emerges via the cooperative social efforts and can be used by others to learn. Moreover, the relevance and speed of knowledge acquisition from these networks may be more efficient than from the networks generated through wide-scale ontological plans and efforts. We apply the techniques of algebraic topology of graphs [18–22] to investigate higher-order structures that characterise the connection complexity between knowledge elements in the emergent networks. Specifically, we aim to determine

- the metrics to quantify the higher-order combinatorial structures which contain the logical units of knowledge as the actors use them in communication;
- the role of innovative contents brought over time by the experts in building the network architecture.

In addition to the standard graph-theoretic metrics and community detection in the emergent networks of knowledge units, we describe their hierarchical organisation using several algebraic topology measures. Further, we identify the appearance of new tags over time and investigate the subgraphs (innovation channels) where these new cognitive elements attach to the existing network. By tracking topology measures over the consecutive time periods for the innovation channel together with the topology of the entire network, we quantify the impact of the new-added contents. Our main findings indicate that the networks of cognitive elements map to a nontrivial hierarchical architecture which contains aggregates of high-order cliques. The increasing structural complexity of these networks over time, owing to the innovation expansion, is consistent with the logical structure of knowledge that they contain and temporal correlations in the appearance of new cognitive contents.

In the following, the networks of tags are built from the empirical data for four successive one-year periods. At the initial stage, the networks are filtered to remove redundant links. At the next stage, network measures are obtained at the graph level, and the community structure is determined. At the final stage, the algebraic topology analysis of these networks for different periods and filtering levels is performed. The analysis is focused on the subgraphs, which are related to the appearance of new tags, representing the innovation channels of these networks.

## Emergence of the tags networks

### The Q&A process and structure of the empirical data

In this work, we have constructed knowledge networks from the empirical data, which are collected and described in Ref. [5]. In the data, the knowledge contents are mathematical tags used in the communications on Q&A system *Mathematics Stack Exchange*. In particular, the content of each question is specified (tagged) by one or more (maximum five) tags according to the standard mathematical classification scheme. While in Ref. [5] we investigated the role of expertise in the social process taking part on the co-evolving bipartite network of users-and-questions, here we focus on the network of tags as the elementary units of knowledge that are used by the actors in this process. With the help of the agent-directed modeling, in Ref. [5] we have demonstrated that the considered empirical process obeys the fundamental assumption of knowledge creation, i.e., that at least minimal matching between the contents of the question and the expertise of answering actor occurred in each event. Therefore, the emergent network of tags reflects the way in which these knowledge units are used in the process and, indirectly, the expertise of the social community. Moreover, the architecture of the emergent network of tags is expected to mirror the logical structure of knowledge, as it is presented by the experts involved in the knowledge-creation process.

To be consistent with the previous studies and the associated analysis of Ref. [5], we use the same dataset that was downloaded on May 5, 2014, from https://archive.org/details/stackexchange and contains all user-contributed contents on Mathematics since the establishment of the site, July 2010, until the end of April 2014. Specifically, the considered dataset contains 269818 questions, posted and answered by 77895 users, 400511 answers, and 1265445 comments. For the present analysis, from the available high-resolution data we use the information about questions, i.e., ID of each question, its content as a list of tags, and time stamp. The tags and their combinations define the knowledge landscape whose size is not constant but increases with time and the number of posted questions. In this way, the innovation increases as the key feature of the collective knowledge creation [5]. By investigating the network of tags, here we examine how the knowledge creation can be expressed by the topological complexity of the expanding knowledge landscape.

*Mapping data to networks of tags* is performed within four consecutive periods; a period is one-year long. First, the questions that are posted within the considered year period are selected, and a unique set of tags that are involved in these questions is formed. Each tag represents a node of the tags network. Two tags (*i*, *j*) are linked by multiple connections *w*_*ij*_, where the link multiplicity *w*_*ij*_ = 0, 1, 2, ⋯ represents the number of common questions in which the considered pair of tags appeared in the selected dataset. The resulting networks are termed tagNetY-k, where *k* = 1, 2, 3, 4 indicates the considered year period.

### Graph measures of tags networks without redundant connections

**Fig 1:**
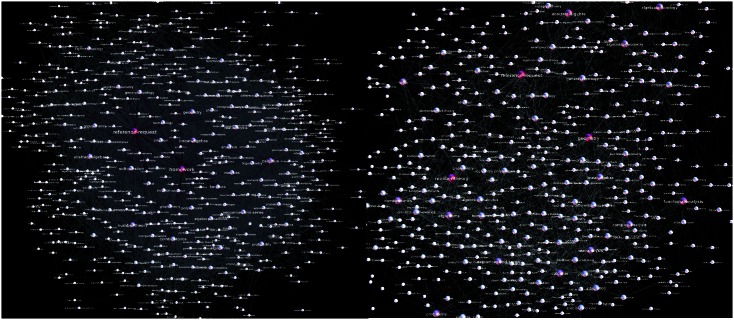
The network tagNetY-1: a close-up of unfiltered network near some large nodes (left) and the whole network filtered at confidence level *p* = 0.1 (right).

The raw networks of tags contain a large number of redundant connections leading to a large-density graph, cf. an example in Fig 1. To move forward, we first apply an advanced procedure to eliminate the potentially redundant links.

*Filtering redundant connections in a network of tags* is motivated by the following facts. In the data, the number of tags is between 500 and 1000 while the number of posted questions per year are between 15 and 120 thousand, which results in a quite dense network of tags. On the other hand, a broad distribution of the tags frequencies [5] suggests that a relatively small number of tags occurs quite frequently. Among the most frequent tags are “homework”, “proof-writing”, “reference-request”, and “terminology”, which are not related to any particular field of Mathematics but rather determine the type of question asked. For this reason, these tags can occur in many different combinations of tags, thus increasing the network’s density. Here, we apply an algorithm to decrease the network’s density by identifying the edges that do not incur as a result of a random process. For this purpose, the weighted network is considered as a multigraph where the weight *w*_*ij*_ represents a multiplicity of links between the pair of nodes (*i*, *j*). We apply the filtering technique described in Ref. [23]; it utilizes a random configurational model for weighted graphs that preserves the total weight of the realised links, *W* = ∑_*k*_ *s*_*k*_, as well as the node’s strength *s*_*k*_ = ∑_*j*_ *w*_*ij*_ on average. To avoid the influence of the filtering on higher structures, we apply the algorithm to each link independently.

A pair of nodes (*i*, *j*) is selected proportionally to their strengths *s*_*i*_ and *s*_*j*_. In the considered network, the selected pair is connected by the weighted link of the multiplicity *w*_*ij*_. In the random configurational model, the occurrence of a link with multiplicity *m* between the selected pair of nodes is given by the conditional probability

Pij(m|si,sj,W)=Wmsisj2W2m1-sisj2W2W-m. (1)

Then the probability that the realised weight *w*_*ij*_ of the link (*i*, *j*) occurred by chance (*p*-value) according to the marginal distribution given by Eq (1) is computed as [23]

$$\begin{array}{c} P_{r}\left(w_{ij}\right)=\sum_{m\geq w_{ij}}P_{ij}\left(m|s_{i},s_{j},W\right). \end{array}$$ (2)

The links for which the probability *P*_*r*_(*w*_*ij*_) appears to be larger than a preset confidence level *p* are removed. The remaining edges, which satisfy the condition *P*_*r*_(*w*_*ij*_) ≤ *p*, represent the filtered network with the specified confidence level. Here we examine the structure of the filtered networks obtained for several values of the parameter, *p* ∈ {0.1, 0.05, 0.01}. As an example, the right panel in Fig 1 shows the first year network after the filtering procedure with the confidence level *p* = 0.1.

**Fig 2:**
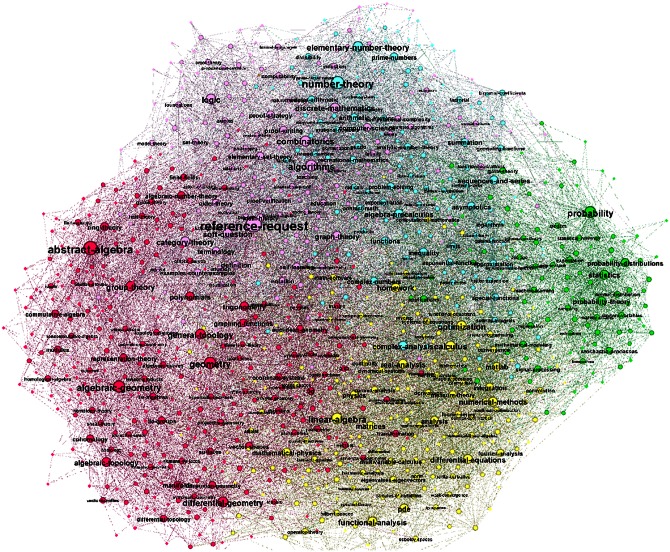
The community structure of the network of tags for the fourth period, which is filtered at *p* = 0.1. In each community, the mutually connected cognitive contents (mathematical tags) are indicated by the nodes’ labels.

The networks of tags for different periods and filtered at various confidence levels are analysed by algebraic topology techniques, as presented in the following Sections. In this regard, we turn the weighted networks into binary graphs, which retain all important topological features of the weighted graphs while making the computation less demanding. Here, we first show that the filtering process leads to a reduced-density graph but preserves the relevant (nonrandom) connections. Specifically, the thematically connected groups of nodes (cf. labels of nodes in Figs 1 and 2) appear to form distinct communities on the network. In these networks, mostly non-overlapping communities occur. Consequently, they are suitably identified by methods based on the optimisation of the modularity [24–26]. A module is recognised as a densely connected group of nodes that are sparsely connected to nodes in other groups [27]. For a better comparison of different networks, the communities are systematically determined at the same resolution parameter (standard resolution 1.0 in Gephi, the open graph visualization platform http://gephi.org). This large-scale clustering of the knowledge networks appears systematically during the network growth. See also the structure of innovation channels studied in the following Section.

For comparison, in Table 1 we summarise the standard graph-theoretic measures [27] of the networks of tags for four consecutive periods and the confidence level *p* = 0.1. Note that the network of tags grows over years by the appearance of new tags, but also shrinks by the number of tags that appeared in the previous period and were not used in the current period.

**Table 1:** The graph-level measures for tags networks for four consecutive periods, filtered at confidence *p* = 0.1.

Net	*N*	〈*k*〉	〈*ℓ*〉	*d*	Cc	*ρ*	*M*
TagNetY-1	582	10.07	3.02	6	0.365	0.018	0.439
TagNetY-2	702	14.45	2.86	5	0.365	0.021	0.441
TagNetY-3	856	20.09	2.72	5	0.351	0.023	0.436
TagNetY-4	1033	22.52	2.68	5	0.338	0.022	0.422

The number of nodes *N*, average degree 〈*k*〉, average path length 〈*ℓ*〉, diameter *d*, clustering coefficient Cc, graph density $\rho =\frac{L}{N(N-1)}$, and modularity *M* = ∑_*i*_ (*e*_*ii*_ − (∑_*j*_ *e*_*ij*_)^2^), where the summation runs over different communities.

## Topology of the tags networks

In addition to the standard graph-theoretic analysis, cf. Table 1, we apply techniques of algebraic topology to determine simplices and simplicial complexes, which describe higher order structures of these networks. Definitions and detailed explanation of topological quantities used in this presentation may be found in Ref. [19] and references within. The simplices are identified as maximal cliques of all orders, i.e. dimensions. Then the topological complexity of the simplicial complex constructed from the complex network is quantified by the number of cliques at each topological level (dimension) *q*, starting at *q* = 0 up to the *q*_*max*_ − 1. A clique at level *q* = 0 is an isolated node while *q* = 1 is a link, *q* = 2 is a triangle and so on up to the level *q*_*max*_ − 1 representing the largest clique found in the network.

### Algebraic topology measures

We use the Bron-Kerbosh algorithm [21, 22] to determine cliques of all orders that are present in the studied network. The resulting matrix of maximal cliques (MC) thus contains information about the identity index of each clique as well as the identity index of each node that participates in that clique. Using rich information of the MC matrix, we can characterise the topological spaces around each node as well as the organisation of cliques in the entire network at each topological level. These goals are achieved by determining several node-related quantities [19] in addition to the commonly defined structure vectors of the network [18–20, 28].

In particular, the topology vector **Q**^**i**^ is associated with the node *i*

$$\begin{array}{c} Q^{i}=\left\{Q_{q_{max}-1}^{i},Q_{q_{max}-2}^{i},\cdots ,Q_{0}^{i}\right\}\;, \end{array}$$ (3)

where the components $Q_{k}^{i}$, *k* = 0, 1, ⋯*q*_*max*_ − 1, describe the number of *k*-dimensional cliques in which the node *i* participates. Then the influence of a node in the overall network architecture is quantified by *topological dimension* *dimQ*^*i*^ of the node *i*, which is introduced in [19]; it is defined as the total number of all cliques in which the node *i* participates

$$\begin{array}{c} dimQ^{i}=\sum_{q=1}^{q_{max}-1}Q_{q}^{i}. \end{array}$$ (4)

**Fig 3:**
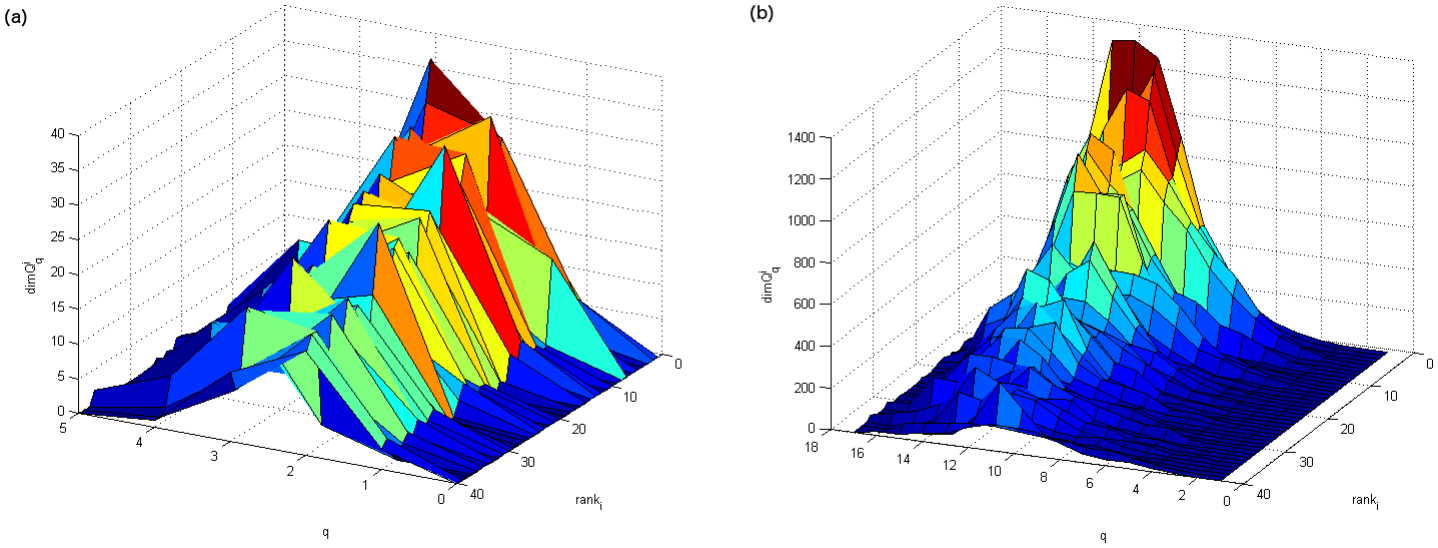
Components $Q_{q}^{i}$ of the first 40 tags ranked by their topological *dimQ*^*i*^ for the tagNetY-1 network filtered at *p* = 0.1 (a) and with no filtration (b).

To demonstrate the relevance of nodes, we compute the topological dimension of each node in the original and filtered network of tags for the first-year interval, which are shown in Fig 1. The components at each *q* level of the top 40 nodes (tags), ordered according to their topological dimension, are displayed by three-dimensional plots in Fig 3. As this figure shows, the applied elimination of the links reduces not only the node’s topological dimension but also changes the structures at *q*-levels where the considered node is present. Consequently, the ranking order of a particular node can be changed (see the corresponding lists of nodes in Table 2), which is compatible with the altered importance of that node in the filtered network.

**Table 2:** Names of the first twenty tags ordered according to their topological dimension in the network of tags before filtering and after filtering at the indicated confidence level *p* has been performed.

before filtering	*p* = 0.1	*p* = 0.05	*p* = 0.01
calculus	number theory	number theory	geometry
linear algebra	geometry	geometry	number theory
analysis	algebraic topology	functional analysis	calculus
homework	combinatorics	sequences and series	algorithms
reference request	abstract algebra	combinatorics	functional analysis
probability	functional analysis	algebraic topology	abstract algebra
sequences and series	algebra precalculus	abstract algebra	reference request
geometry	group theory	differential geometry	real analysis
functions	real analysis	calculus	algebra precalculus
real analysis	differential geometry	real analysis	logic
combinatorics	logic	probability	sequences and series
abstract algebra	sequences and series	algebraic geometry	probability
number theory	soft question	algebra precalculus	linear algebra
terminology	probability	algorithms	algebraic topology
complex analysis	integration	soft question	combinatorics
general topology	algorithms	logic	complex analysis
category theory	complex analysis	analysis	dierential geometry
algebraic geometry	analysis	complex analysis	soft question
discrete mathematics	differential equations	integration	discrete mathematics
logic	calculus	differential equations	analysis

**Fig 4:**
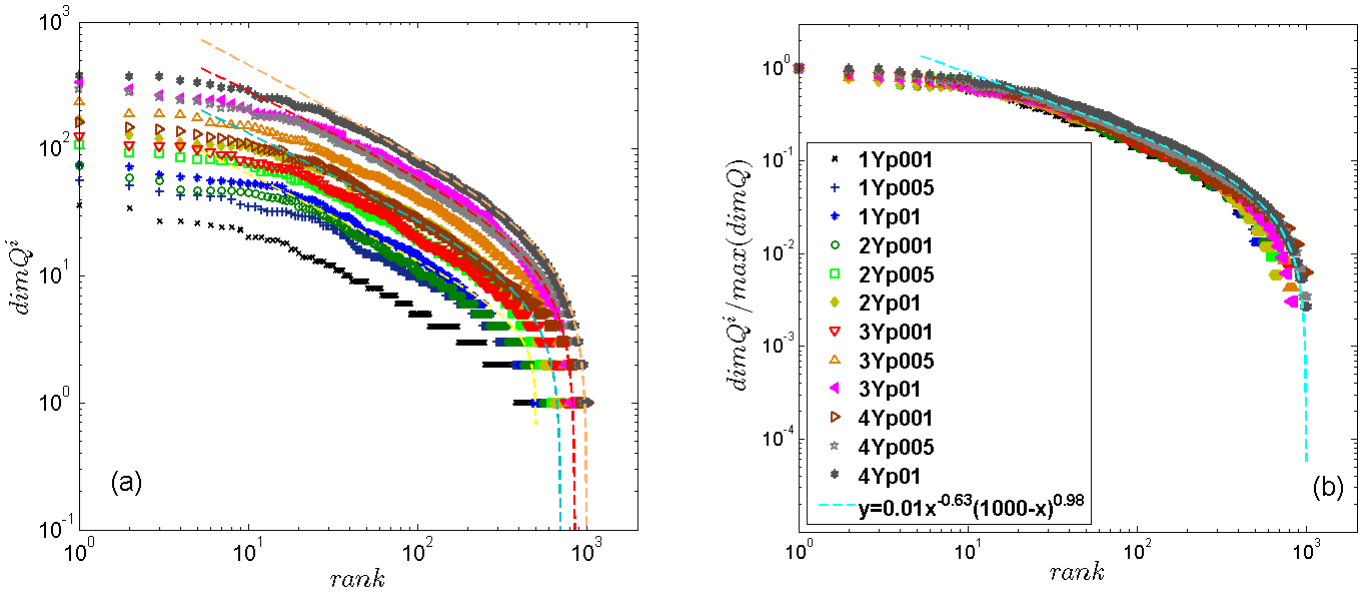
Ranking distributions of the topological dimension of tags *dimQ*^*i*^ for all years and all *p* values (a) and scaled distribution *dimQ*^*i*^/*max*(*dimQ*^*i*^) of all data (b). The legend abbreviations: 1Yp001 indicates the first-year network filtered at the level *p* = 0.01, and so on. Fit lines are according to the discrete generalised beta function (5); in panel (a) the parameter *b* = 0.67 ± 0.03 and *c* varies from 0.32 for 1Yp001 and 0.71 for 2Yp001 to 0.82 for 3Yp001 and 4Yp001, with error bars ±0.03.

We further compare the role of individual nodes in the networks evolving over time, which are filtered at different confidence levels, i.e., *p* = 0.1, *p* = 0.05 and *p* = 0.01. We determine the topological dimensions of all nodes in the corresponding filtered networks for the four successive year-periods. The ranking distributions of the node’s topological dimensions are displayed in Fig 4a. This Figure shows that, first, nodes with a gradually higher topological dimension appear at later periods, suggesting that topological complexity of tags networks increases over years. Furthermore, within each year, the reduced confidence level *p* results in a simpler structure of the nodes’ neighbourhood (and possible shifts in the ranking order of nodes, as mentioned above). However, all networks exhibit a broad ranking distribution of the node’s topological dimension with a power-law section. The distributions are fitted by the discrete generalised beta function

$$\begin{array}{c} f\left(x\right)=ax^{-b}{\left(N+1-x\right)}^{c} \end{array}$$ (5)

with different parameters *a*, *b* and *c*. The robustness of the observed scaling feature is further confirmed by the scaling collapse of all curves to a master curve, shown in Fig 4b. The scale-invariant ranking, where the node’s topological dimension is scaled by the maximal dimension found in the corresponding network, suggests that the relative topological complexity of the tags networks is preserved over time and the degree of filtering.

### Topological spaces in the filtered networks of tags

**Fig 5:**
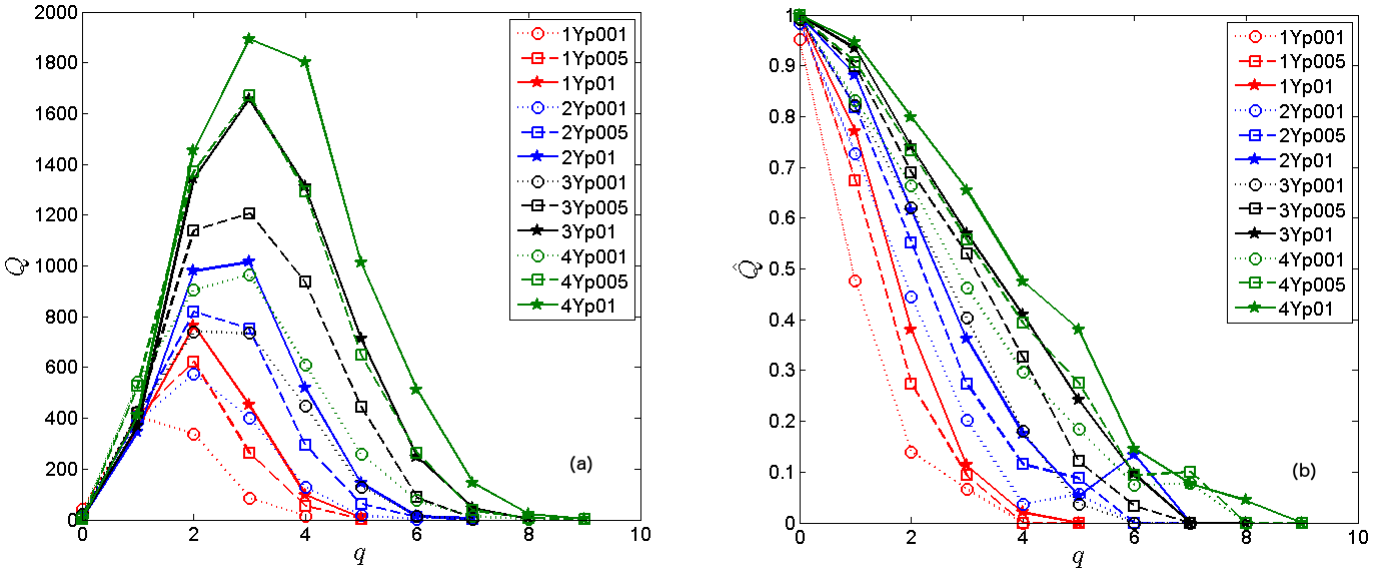
The components of (a) the first structure vector *Q*_*q*_ and (b) the third structure vector ${\hat{Q}}_{q}=1-n_{q}/Q_{q}$ plotted against the topology level *q* for each year period and three filtering levels *p* = 0.1, 0.05, 0.01. The legend abbreviations are explained in connection to Fig 4.

To characterise the structure of the topological levels *q* = 0, 1, 2, ⋯*K* of the entire graph, we compute three commonly used structure vectors [18–20, 28, 29]. In particular, the first structure vector

$$\begin{array}{c} Q=\{Q_{q=K},Q_{q=K-1},\cdots ,Q_{q=1},Q_{q=0}\} \end{array}$$ (6)

has *K* + 1 components that describe the number of *q*-connected classes, where *K* + 1 = *q*_*max*_ indicates the size of the maximal clique found in the graph. Furthermore, the components of the second structure vector

$$\begin{array}{c} N_{s}=\left\{n_{q=K},n_{q=K-1},\cdots ,n_{q=1},n_{q=0}\right\} \end{array}$$ (7)

designate the number of simplexes from the level *q* up to the top level. The third structure vector is often defined such that its *q*-level component

$$\begin{array}{c} {\hat{Q}}_{q}=1-\frac{Q_{q}}{n_{q}} \end{array}$$ (8)

determines how the simplices of higher order are connected at the level *q*. Fig 5 summarises the components of two structure vectors for the tags networks emerging over different periods and varied filtering level *p*.

By comparing the curves for different one-year periods but fixed filtering level, say *p* = 0.1, we observe that the network topological complexity increases over time. It manifests in the increased number of connectivity classes (components of the first structure vector) at all topological levels as well as the shift of the maximum from *q* = 2 (triangles), in the first year, to *q* = 3 (tetrahedra) and *q* = 4 (5-cliques), in the fourth year. At the same time, we observe that the number of topological levels increases as well as the connectivity among the cliques at each topology level, cf. the third structure vector in the Fig 5b.

On the other hand, by decreasing the filtering confidence level *p*, a more sparse network is obtained having a smaller number of topological levels and a reduced number of simplicial complexes. However, they proportionally preserve the above-described tendency of the enhanced complexity of combinatorial spaces over time. The corresponding curves for *p* = 0.05 and *p* = 0.01 are also shown for each year-period in Fig 5. According to the structure vectors in Fig 5, all filtered networks exhibit a systematic shift towards richer topology in later years. Once again, these results confirm the structural stability in Fig 4 of the emergent networks of tags, which complements the logical organisation of knowledge contents in the communities in these networks, demonstrated in Fig 2 and in the following Section.

## Clustering of the innovative contents

### Three aspects of innovation in the knowledge creation

The innovation growth [5, 30] is a crucial element of the process of knowledge creation. In the voluntary system, the innovation that comes from the expertise of the actors involved in the process was shown [5] to expand the knowledge space over time. To quantify the impact of innovation onto the architecture of the emerging knowledge networks, we consider the following three aspects of the innovation:

- the appearance of new tags due to the actor’s expertise;
- the occurrence of new combinations of tags expanding the knowledge space;
- the emergence of new combinatorial topological structures enriching the architecture of the knowledge network.

In the following, we discuss in detail these features of innovation.

**Fig 6:**
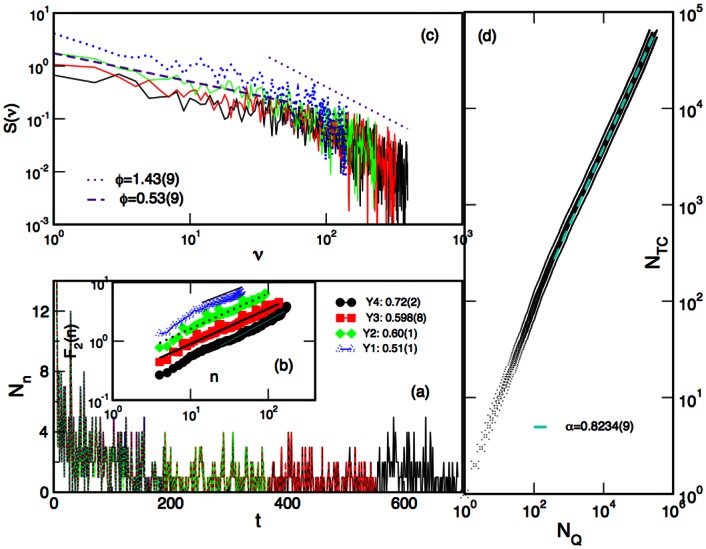
The temporal sequence of the appearance of new tags present in the networks for Year-1, Year-2, Year-3 and Year-4 periods (a). Temporal resolution is two days. The scaling of the standard fluctuation function (b) and the power spectrum (c) of these time series. Panel (d) displays increase in the number of new combinations of tags as a function of the number of questions over time.

Fig 6a contains time sequence of the first appearance of tags that are present in the data of each one-year period. Naturally, the sequence for Year-1 is the shortest, while the sequence for Year-4 is the longest, since some tags that are present in Year-4 appeared in the earlier periods. The time series contains the number of new tags appearing in the sequence of two-day time intervals. The fractal analysis of these time series and their power spectrum, shown in Fig 6b and 6c suggest that the appearance of new tags is not random but exhibits long-range temporal correlations. Specifically, the plots in Fig 6b represent the fluctuation function *F*_2_(*n*) of the standard deviations of the integrated time series at the interval of length *n*. They reveal scaling regions (of different length for each time series) which permit determination of the Hurst exponent via *F*_2_(*n*)∼*n*^*H*^. Values of the Hurst exponent *H* indicated in the legend suggest the fractal structure of the fluctuations. It appears that the fractality increases over time from nearly random time series with *H* = 0.51 ± 0.01 in Year-1, to strongly persistent fluctuations with *H* = 0.72 ± 0.02, in Year-4.

Similarly, power spectra of these time series in Fig 6c exhibit long-range correlations according to *S*(*ν*) ∼ *ν*^−*ϕ*^ with two distinct exponents in high and low frequency regions. While the low-frequency feature is similar for all considered periods, the pronounced scaling in the high-frequency region gradually builds over years.

The number of unique combinations of tags was examined in the whole dataset and plotted against the number of posted questions in Fig 6d. The plot exhibits a power-law behaviour $N_{TC}∼N_{Q}^{\alpha }$ in the range above 10^2^ posted questions. It represents the Heaps’ law which appears to be in agreement with the ranking distribution of frequencies of the unique combinations of tags, i.e., the Zipf’s law, as discussed in [5]. The occurrence of Heaps’ law is a manifestation of the innovation growth [5, 30] in the process of Q&A. The exponent *α* < 1 indicates a sublinear growth of innovation with the number of posted questions. This dependence suggests that a fraction of displayed items brings new combinations of tags while the remaining questions use the already identified combinations.

### The structure of innovation subgraphs

**Fig 7:**
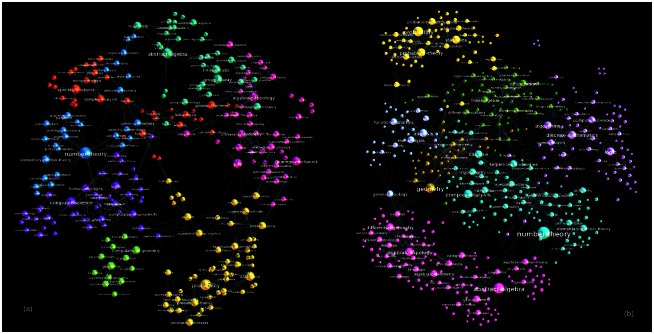
The structure of the innovation channel at the beginning of Year-2 (left) and Year-3 (right). New tags were added to the filtered tags network of the previous year, forming structures of higher dimension than a triangle. Communities of well-connected nodes show the logical grouping of mathematics subject categories, indicated by labels on nodes.

The appearance of new tags in the Q&A process leads to the expansion of the knowledge network. In particular, the network grows by the addition of new nodes (cf. Table 1), as well as by increasing its topological complexity measured by the presence of simplicial complexes of a high order. In the remaining part of this section, we investigate how the new tags attach to the existing nodes and affect the formation of higher order structures in the knowledge network. For this purpose, we first define an *innovation channel* as a subgraph related with the new tags appearing at the end of a considered one-year period. Specifically, the subgraph in the network (filtered at *p* = 0.1) contains newly added tags together with the tags to which they attach and form simplices larger than a single link (i.e., triangle or higher dimensional structure). The two plots in Fig 7 show the structure of the innovation channels at the beginning of Year-2 and Year-3 periods, respectively.

The innovation channels in Fig 7 grow over a one-year period; moreover, the innovative nodes stick with the rest of the network (previously existing nodes and links) making with them a tight structure that involves higher-order combinatorial spaces up to the largest clique. The community structure in the innovation subgraphs, which is demonstrated in Fig 7, reflects the thematic grouping of the entire knowledge network, as presented in Fig 2. For example, the newly added tag “cohomology” sticks to the group where we also find “algebraic topology”, “differential geometry”, “abstract algebra”, “complex geometry” and other thematically related tags, cf. the lower left community in Fig 7 right panel. On the other hand, the added tag “computational complexity” links to the community with “discrete mathematics”, “algorithms”, “logics”, “combinatorics”, “computer science” and others, cf. the rightmost community in the same Figure. Similarly, the node labels in all identified communities confirm their thematic closeness. Therefore, the expansion of the knowledge network by the addition of innovative contents systematically obeys the overall logical structure of (mathematical) knowledge. As mentioned earlier, the core of this feature of knowledge creation lies in the crucial role of the actor’s expertise in the process of meaningful cognitive-matching actions. The logical structure of individual knowledge of each actor gets externalised during the process of Q&A.

**Fig 8:**
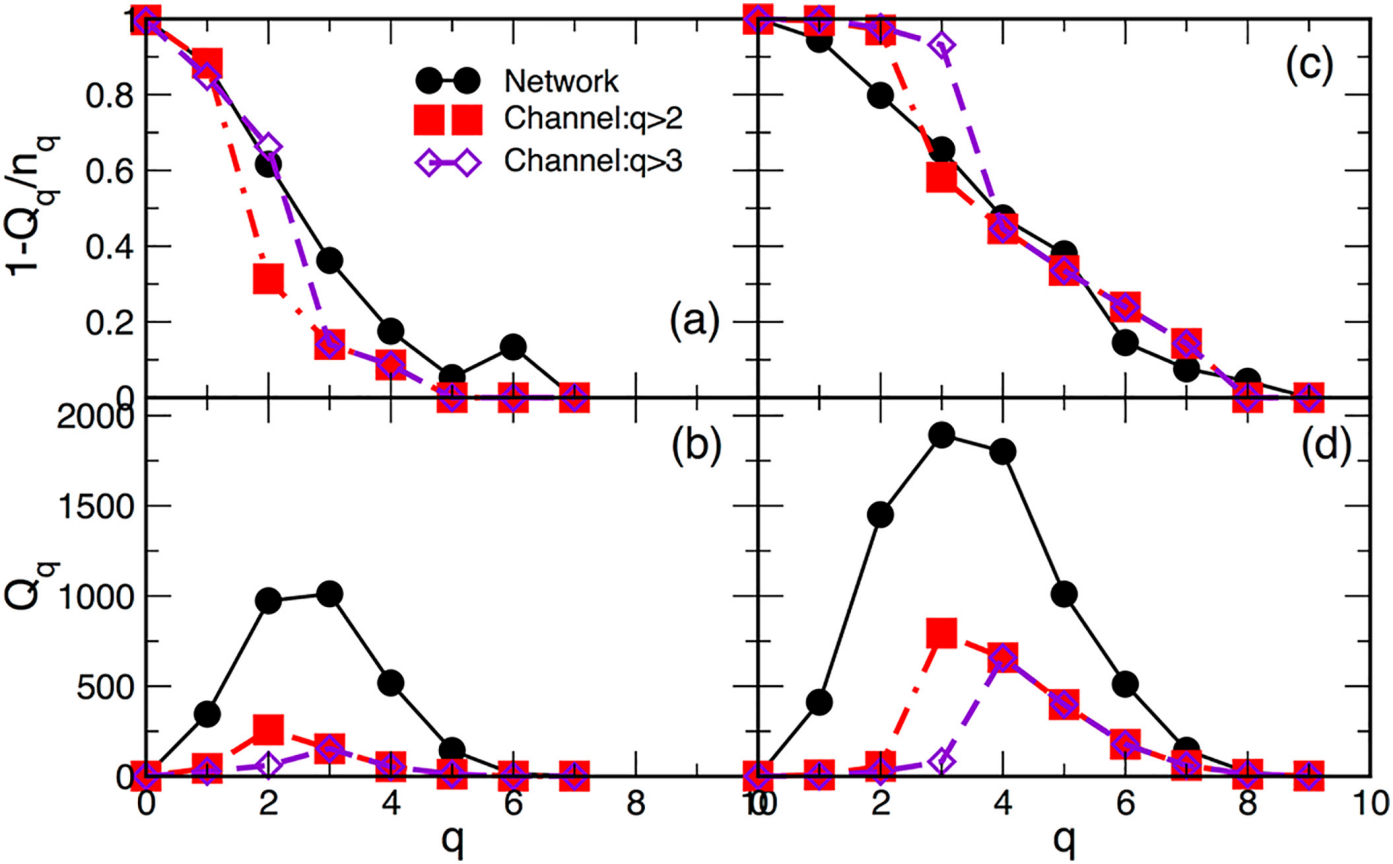
(a) and (c) The third structure vector and (b) and (d) the first structure vector for the networks of tags in Year-2 (left panels) and Year-4 (right panels) and the corresponding innovation channels above the level *q* = 2 and *q* = 3.

**Fig 9:**
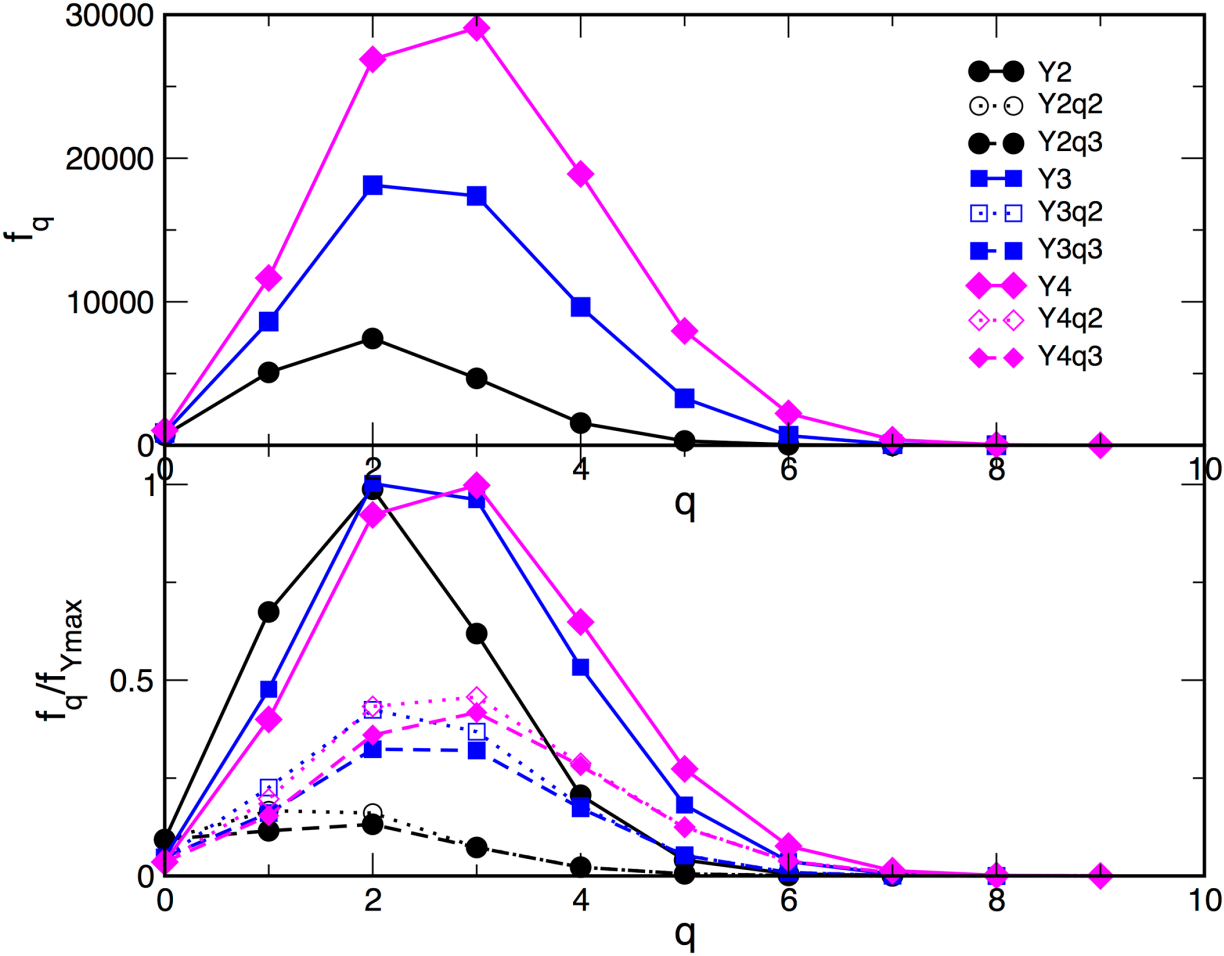
Response *f*_*q*_ plotted against the topology level *q* for networks of Year-2, Year-3, and Year-4 (top panel) and for the corresponding innovation channels scaled with the year maximum value (bottom panel).

According to the results in Fig 6, the appearance of innovative contents boosts the process of knowledge creation, leading to the observed temporal correlations, characteristic of collective dynamics. Analogously, here we show that the structure of innovation channels enriches the topological spaces of the knowledge network. In Figs 8 and 9 we summarise the topological measures of the innovation channels and compare them with the corresponding measures of the entire network. In addition to the structure vectors defined in Eqs (6)–(8), here we also consider the topological “response” function *f*_*q*_ to express the shifts in the topology at each level *q* in response to the changes in the network size. Formally, *f*_*q*_ is defined [20] as the number of *simplices and shared faces* at the level *q*.

Interestingly enough, the third structure vectors in Fig 8a and 8c show that the corresponding channels exhibit a better connectivity up to the level *q* = 4 of 5-clique than the background network. This feature of the innovation channels suggests the leading role of the innovative tags in the observed increase of the topological complexity of the network over years. This conclusion compares well with the number of connectivity classes at different topological levels, namely the first structure vectors in Fig 8b and 8d. The topology of the channel determines the most ubiquitous structure in the entire network, corresponding to the peak in the first structure vector. Furthermore, the increase of the topological complexity of the knowledge graphs over consecutive periods is illustrated by the topological “response” function *f*_*q*_, which is shown in Fig 9. It manifests in the increase of the number of topology levels, as well as the number of simplices and shared faces at each topology level. Also, the maximum of the function *f*_*q*_ shifts towards more complex structures, i.e., from triangles at Year-2 to tetrahedra in Year-4. As the plots in the lower panel of Fig 9 show, these topological shifts in the networks of different periods are tightly reflected in the structure of the corresponding innovation channels.

## Conclusions

Information processing underlines the evolution and structure of various social networks [31–33]. The creation of knowledge through questions-and-answers requires meaningful interactions with the actor’s expertise adjusted to the needs of other participants; consequently, it leads to the accumulation of the sound knowledge and the expansion of knowledge space [5]. In the studied example, we have demonstrated how the algebraic topology measures can characterise the connection complexity of the emergent knowledge networks. Using the data of questions-and-answers from the Stack Exchange system Mathematics, we have shown how the network of mathematical tags, as constitutive elements of knowledge, appears and evolves with the actor–question–actor-answer interactions over time.

The connections among different tags reflect their use by the actors possessing the expertise, which (at least partially) overlaps with the contents of the considered question. The networks of tags are filtered by removing the extra edges which may have appeared by chance with a specified confidence level. We have applied the filtering at the level of (uncorrelated) edges to preserve the higher-order structures, which have been the focus of this study. Our results reveal that the process preserves the genuine structure of knowledge networks consisting of thematically connected tags communities. For example, five communities in Fig 2 appear in the filtered network of tags in Year-4. Considering the higher-order topological spaces, the filtered networks of tags exhibit a robust structure. The hierarchy of nodes sorted out according to their suitably scaled topological dimension is represented by a unique curve, independent of the evolution time and the filtering level.

The appearance of new contents (tags) over time plays a significant role in the process of knowledge creation and the related networks. As it was shown in [5], the occurrence of new contents and new combinations of contents are chiefly related to the expertise of newly arriving users. Therefore, the introduced combinations of tags obey the logical structure as it is presented by the participating experts. The growing number of unique combinations leads to the advance of innovation [5], as also shown in Fig 6d. Moreover, their appearance is conditioned by the cognitive-matching interactions and the user’s activity patterns. These features of the social dynamics are manifested in the non-random (persistent) fluctuations and long-range temporal correlations, as demonstrated in Fig 6a, 6b and 6c. Further, the performed algebraic topology analysis has revealed the role of these innovative contents in building the architecture of knowledge network. Specifically, we have found that:

- the newly appearing tags connect to the current network at all levels from a single link to the cliques of the highest order;
- the innovation channel is recognised as a subgraph containing simplices larger than or equal to a triangle in which at least one of the new tags occurred; its growth and the increased topological complexity over time provides the evolution pattern of the entire network;
- the growth of the innovation channel is consistent with enhanced fractal features and temporal correlations of the appearance of new contents over time; it systematically obeys the sensible connections of contents, as also demonstrated in Fig 7.

The presented results reveal that the creation of new combinations of knowledge contents (or innovation) is compatible with the non-random correlations in the sequence of new contents and their linking to the knowledge network. Hence the innovation expansion, as a core of each knowledge-creation process, can be additionally quantified by the fractal features of time series of new tags as well as the algebraic topology measures of the network’s innovation channel. Hidden beneath these quantifiers of the emergent knowledge networks is the dynamics of human actors and their expertise, which provides the logical structure of the collective knowledge. Our approach consists of the appropriate data filtering, fractal analysis of time series, and algebraic topology techniques applied to the emergent knowledge networks and their innovative channels. The methodology can be useful to the analysis of a wide class of networks where the actors and their artefacts, as well as the cognitive elements used in the process, are clearly identified. These may include, among others, networks created by science, engineering, business and economics communities based on online collaborations. Further, such examples may also include a collection of articles (e.g. journal articles) referring to each other, where their logical units are marked. In some such situations, keywords, memes, and concepts can be identified by machine learning methods.
